# Recent Advances in Electrochemical Biosensors for the Detection of Milk Adulterants

**DOI:** 10.3390/bios16020092

**Published:** 2026-02-02

**Authors:** Roopkumar Sangubotla, Anthati Mastan, Jongsung Kim

**Affiliations:** 1 Department of Chemical, Biomolecular, and Battery Engineering, Gachon University, 1342 Seongnam Daero, Seongnam-si 13120, Republic of Korea; gachonroop@gmail.com; 2 Department of Microbiology and Botany, School of Sciences, Jain (Deemed to be University), Bengaluru 560027, Karnataka, India; mastan.anthati@gmail.com

**Keywords:** milk adulterant detection, optical biosensors, electrochemical sensors, wearable food sensors, adulteration monitoring, food safety analytics

## Abstract

The precise and reliable detection of milk adulterants has garnered increased scientific interest owing to the rising incidence of food fraud. Recent years have witnessed substantial advancements in optical and electrochemical biosensors for the quick, sensitive, and on-site determination of adulterants. This review thoroughly emphasizes recent developments in electrochemical biosensors, encompassing amperometric, voltammetric, impedimetric, and photoelectrochemical sensors, alongside optical biosensors such as colorimetric, fluorometric, and plasmonic systems. Significant focus is directed towards determination of critical milk adulterants, including variations in pH, urea, formaldehyde (FA), melamine (MEL), nitrates (NO_3_^−^), nitrites (NO_2_^−^), and sulfites (SO_3_^2−^). The sensing mechanisms, functional nanomaterials, analytical efficacy, and sample-handling techniques of the described biosensors are critically examined. Moreover, key challenges regarding matrix interference, sensor stability, reproducibility, regulatory validation, and large-scalability are addressed. Ultimately, future directions towards economical, portable, wearable, and Internet of Things (IoT)-integrated biosensors for continuous dairy monitoring are discussed, highlighting the necessity for standardized validation protocols and next-generation technologies in food safety.

## 1. Introduction

Milk and dairy products are indispensable components of the human diet, valued for their high nutritional content, energy density, and ability to replace less nutritious food sources. Beyond their dietary importance, the dairy sector plays a pivotal role in global economies, sustaining livelihoods and contributing significantly to agricultural output. The intricate dairy matrix enhances nutrient absorption and bioavailability, while milk adulterants, bioactive compounds, and carbohydrates contribute to growth, immunity, and metabolic regulation. Milk supplies 28 of 29 essential nutrients, including high-quality proteins, calcium, and vitamins, thereby serving as a leading dietary source of human nutrition. Globally, milk production exceeds 800 million tons annually, and its versatility, minimal processing requirements, and deep cultural roots make it an irreplaceable dietary element. Adulterated milk consumption is increasingly associated with substantial public health consequences that extend beyond simple nutritional dilution to the immunomodulatory effects [1]. Recent studies indicate that illicit adulterants, including melamine (MEL), urea, and other chemical pollutants, compromise the nutritional quality of milk and present significant health risks to consumers. For instance, exposure to MEL from contaminated milk products has been linked to renal failure and various kidney disorders. The inclusion of urea and other compounds, alongside MEL, undermines physiological activities, potentially leading to gastrointestinal distress and systemic diseases with prolonged use. The presence of adulterants in milk poses significant food safety concerns and may adversely affect human health, emphasizing the importance of reliable and sensitive analytical detection [2,3,4,5]. However, milk adulteration and protein quality assessment remain persistent challenges affecting both safety and consumer trust. The absence of standardized analytical protocols and variations in sample preparation hinder reproducibility in quality control [6]. Despite progress in proteomic and chemometric methods for detecting fraudulent samples, species-specific identification often remains difficult due to conserved peptide sequences in processed dairy products. Moreover, milk adulteration leads not only to economic loss but also to health risks, particularly for individuals with allergies to certain proteins. Techniques such as MALDI-MS have shown promise for rapid protein profiling, yet accurate quantification still requires robust calibration models [7].

To address these challenges, biosensors, notably optical and electrochemical sensors, have emerged as rapid, accurate, and portable alternatives for milk quality monitoring. Unlike conventional laboratory assays, biosensors offer real-time, on-site, and highly specific detection of adulterants [8]. Among optical methods, colorimetric and fluorometric sensors have demonstrated significant utility. The colorimetric approach, often based on localized surface plasmon resonance (LSPR) in gold nanobipyramids (AuNBPs), enables the visual detection of adulterants such as urea through distinct color shifts. Complementary fluorometric systems employing Förster resonance energy transfer (FRET) between AuNBPs and upconversion nanoparticles (UCNPs) further improve detection sensitivity and reliability, achieving low limits of detection (LOD) for adulterants [9]. These optical sensors are particularly attractive for their simplicity, speed, and adaptability to portable formats [10]. Parallel advances in electrochemical sensing, which measures current, voltage, or impedance changes in response to analyte binding, have enabled highly selective detection of milk adulterants and proteins, even in complex matrices. Additionally, holographic sensors have recently been explored for real-time, wearable milk quality monitoring, representing the state-of-the-art strategy for continuous and on-site food safety assessment [11]. Despite these advances, significant challenges persist in miniaturization, power efficiency, data security, and regulatory approval. Yet, the translation of such biosensing systems into wearable devices offers immense potential in both public health monitoring and personalized nutrition management.

Apart from chemical adulteration, several factors affect milk quality and authentication, which ensure the health safety of consumers. The detection of biological pollutants, such as bacteria, as target analytes is not covered in this review, although they are acknowledged as significant food safety problems. The accurate identification of chemical adulterants in milk samples remains difficult due to their low quantities and intricate interference from proteins, lipids, and other endogenous substances. Traditional analytical methods for identifying chemical adulterants are frequently labor-intensive, time-consuming, and require skilled personnel, leading to difficulty in rapid on-site monitoring. In this regard, biosensor-based approaches, especially using nanomaterials and selective recognition components, have become viable substitutes for the sensitive, quick, and practical detection of chemical adulterants in milk [12,13,14,15].

Among nanomaterials, carbon quantum dots (CQDs) have shown exceptional promise in enhancing food safety applications [16]. Carbon quantum dots exhibit remarkable water solubility, chemical stability, and tunable fluorescence, enabling their use in food packaging, microbial detection, and adulteration sensing [17]. Their structure, surface passivation, and synthesis route significantly influence their optical and chemical properties, and the use of sustainable, low-cost raw materials in CQD synthesis aligns with environmentally conscious sensor development [18]. Carbon quantum dot-based fluorescence quenching systems utilizing FRET, photoinduced electron transfer (PET), or inner filter effect (IFE) have facilitated rapid food analysis, though scalability and reproducibility still limit their commercialization [19,20].

The advantages and disadvantages of previously reported optical and electrochemical biosensors were discussed in this review in a more systematic way. Furthermore, significant mechanisms, sample preparation, and interference effects, as well as storage stability, were explained in detail in comparison with reference methods for both the optical and electrochemical biosensors. The biosensor performance was studied by employing regulatory thresholds for detecting miscellaneous milk adulterants in a detailed manner. In addition, long-term stability, reproducibility, and batch-to-batch consistency of the different sensors were thoroughly discussed. A separate section was dedicated to wearable and IoT-enabled biosensors for continuous dairy quality monitoring. Finally, the constraints, research needs, and future perspectives for various biosensors used in the detection of milk adulterants were reviewed.

## 2. Milk Adulteration Detection for Food Safety

Milk adulteration remains a critical public health concern, particularly in developing nations where regulatory enforcement is often inadequate. It not only compromises the nutritional integrity of milk but also poses serious health risks to consumers. As one of the most frequently adulterated food products, milk is often tampered with to extend shelf life or to artificially enhance its fat and protein content using a variety of chemical agents [21]. Such adulteration practices result in the introduction of toxic substances that can severely affect major organs, including the liver, heart, and kidneys, leading to chronic illnesses or even fatal outcomes [22]. According to a recent Food Safety and Standards Authority of India (FSSAI) survey, nearly 70% of milk samples tested in India were found to be adulterated with substances such as urea, starch, sugar, salt, and detergents, highlighting the widespread nature of the problem [23]. People commonly add these adulterants to mimic higher protein content or enhance texture and appearance, but they pose a significant threat to consumer safety. Therefore, the precise detection of adulterants, including urea, sodium bicarbonate (NaHCO_3_), hydrogen peroxide (H_2_O_2_), soap, detergents, salt, and starch, is essential for maintaining milk quality [24]. Among these, urea contamination has received particular attention due to its severe physiological effects, such as renal failure, urinary tract obstruction, dehydration, and gastrointestinal hemorrhage. Accurate and sensitive detection of urea is therefore critical in food safety, environmental monitoring, and dairy industry quality control [25]. Conventional analytical methods for adulterant detection are often time-consuming, expensive, and instrument-dependent, limiting their applicability for rapid field analysis. As a result, optical and electrochemical biosensors, both enzyme-based and non-enzymatic, have emerged as promising alternatives for detecting a wide spectrum of milk adulterants, including urea, MEL, formaldehyde (FA), nitrite (NO_2_^−^), and sulfite (SO_3_^2−^), offering rapid, cost-effective, and highly specific monitoring capabilities [26,27]. These biosensing technologies represent a significant leap forward in milk adulteration detection, enabling real-time, on-site, and user-friendly screening of milk safety. Their integration into portable or wearable systems could revolutionize routine quality monitoring and help safeguard public health in the global dairy supply chain.

### 2.1. Colorimetric Sensors

**Figure 1:**
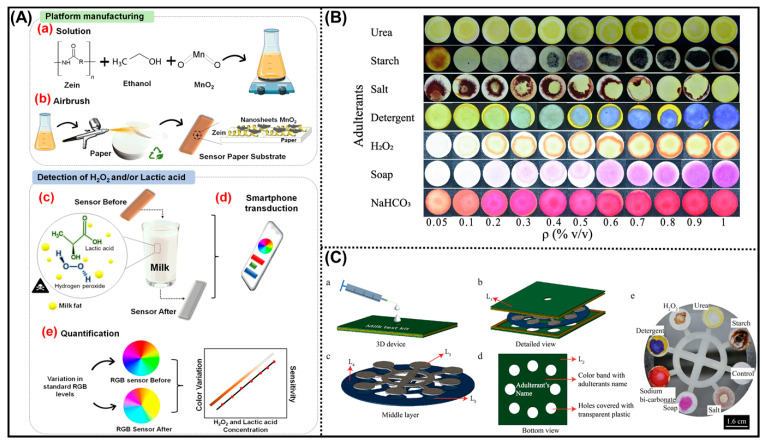
Paper-based sensing platforms for the detection of milk adulterants. (**A**) Development and colorimetric application of a zein–MnO_2_ paper sensor. (**a**) preparation of a zein–MnO_2_ biopolymeric solution; (**b**) deposition of MnO_2_ nanosheets onto paper via airbrush-assisted coating; (**c**) colorimetric response of the sensor toward hydrogen peroxide and/or lactic acid present in milk; (**d**) RGB signal acquisition using a smartphone; and (**e**) quantification of analyte levels based on RGB variations. (Reproduced from Ref. [29]). (**B**) The color changes resulting from the colorimetric reaction at varying adulterant concentrations are shown for seven analytes. (Reproduced from Ref. [32]). (**C**) Three-dimensional paper-based microfluidic device for multi-analyte adulteration testing: Schematic illustration of (**a**) a compact device with top sample inlet, (**b**) layered paper-based microfluidic architecture, (**c**) sandwich assembly with transport and detection zones, (**d**) labeled reference color bands, and (**e**) simultaneous detection of seven adulterants. (Reproduced from Ref. [32]).

Colorimetric sensors have emerged as one of the most efficient, low-cost, and user-friendly analytical tools for detecting milk adulteration. Their operation is based on visually observable color changes triggered by chemical or enzymatic reactions between target adulterants and functionalized nanomaterials [28]. This property makes them especially suitable for on-site, real-time, and resource-limited environments, providing an accessible alternative to conventional laboratory-based techniques. These sensors are particularly effective for identifying common adulterants such as H_2_O_2_, lactic acid, urea, and FA, all of which pose severe health risks when present in contaminated milk [29]. Recent studies have reported significant progress in nanomaterial-based colorimetric sensing platforms (Table 1). For example, Pereira et al. designed a zein/manganese dioxide (MnO_2_) nanosheet composite-based sensor integrated with a smartphone interface, enabling portable and quantitative analysis of milk samples (Figure 1A). The sensor displayed high sensitivity toward H_2_O_2_ and lactic acid, with LOD of 7.2 × 10^−4^ mol L^−1^ and 7.5 × 10^−4^ mol L^−1^, respectively, allowing rapid evaluation of milk freshness and safety. These smartphone-integrated colorimetric platforms are especially attractive for on-site screening and decentralized analysis, as demonstrated for peroxide- and acidity-related milk adulterants. Nevertheless, optical sensors generally exhibit higher detection limits than electrochemical methods and are more susceptible to interference from milk turbidity, background coloration, and ambient lighting conditions, which may limit quantitative robustness.

Similarly, Rao et al. fabricated a flexible, printable titanium oxysulfate/guar gum indicator capable of detecting H_2_O_2_ at concentrations as low as 200 ppm, offering a simple and effective method for on-site milk quality monitoring. Colorimetric approaches have also proven effective for MEL detection, a nitrogen-rich compound illicitly added to mimic higher protein content [30]. The use of non-toxic, biobased components and strong rub resistance supports its suitability for intelligent packaging applications. However, the sensor is single-analyte specific, irreversible, and semi-quantitative, limiting reuse and broader adulterant coverage. Sensitivity remains inferior to electrochemical methods, and matrix effects from milk fats and proteins are not extensively evaluated, which may affect reliability in real sample applications.

Das et al. reported a dual setup using AgNPs and AuNPs, achieving LODs of 1.24 ppm (AgNPs) and 1.68 ppm (AuNPs) in light-dependent resistor (LDR) systems, while the configuration of an ambient light sensor was improved to a sensitivity of 0.64 ppm with a 97% recovery rate [31]. The as-designed plasmonic colorimetric biosensor combined with microcontroller-based LDR and ambient light sensor (ALS) readouts offers notable strengths, including low-cost components, portable instrumentation, rapid response (≈1–3 min), and quantitative MEL detection near regulatory limits. The integration of AgNPs/AuNPs with simple electronics improves quantification compared with naked-eye colorimetric assays and avoids bulky spectrometers. However, the approach relies on extensive milk pretreatment procedures, such as acid coagulation, centrifugation, and filtration), which limits on-site applicability. Moreover, the biosensor is single-analyte specific, irreversible, and sensitive to pH and nanoparticle stability. Additionally, long-term stability, reusability, and batch-to-batch reproducibility are not comprehensively addressed, constraining scalability and routine usage.

Beyond single-analyte detection, multiplexed paper-based devices have advanced the field of colorimetric sensing. Patari et al. introduced a 3D paper-based microfluidic analytical device (μPAD) capable of simultaneously detecting three adulterants at concentrations as low as 0.2% (*v*/*v*) (Figure 1B,C) [32]. The biosensor demonstrates notable strengths, including simultaneous detection of seven milk adulterants, low sample (1–2 mL) and minimal reagent volumes (≈10 µL), rapid response (<30 s), and very low per-test cost (~USD 0.23), making it attractive for resource-limited settings. The device shows good repeatability with a relative standard deviation (RSD) value of <3.5% and reasonable recovery (85–107%). However, the LOD (0.05–0.2% *v*/*v*) remain higher than regulatory residue limits for several adulterants, restricting trace-level monitoring. The approach is single-use, semi-quantitative, and susceptible to reagent evaporation and color-intensity variability, while long-term shelf life and large-scale manufacturing robustness remain insufficiently addressed.

Colorimetric biosensors also exploit enzyme-based reactions for selective adulterant recognition. Shalileh et al. fabricated a paper-based biosensor utilizing halloysite nanotubes (HNTs) loaded with urease and anthocyanin extract as a natural pH-sensitive dye [25]. The hydrolysis of urea by urease leads to an increase in pH, causing a distinct color transition of anthocyanins that can be quantified via smartphone-assisted image analysis within a linear range of 0.5–100 mM. Similarly, Dutta et al. employed citrate-capped AgNPs for detecting urea concentrations between 1.0 and 15 mM in milk with an LOD of 5.56 µM, validated using para-dimethylaminobenzaldehyde (DMAB) as a standard reagent recommended by the FSSAI [33]. The biosensor offered a low-cost, enzyme-free, and visually interpretable approach for urea detection in milk, with acceptable linearity and validation against the FSSAI–DMAB method. However, its performance depends on stability of NPs, requires whey protein preparation, and shows moderate recovery variability at low concentrations, which may limit robustness for routine field deployment.

Further innovations include colorimetric methods for detecting FA and other chemical adulterants. Veríssimo et al. developed an optical fiber-based sensor coated with a PVC/NPOE membrane containing polyoxometalate compounds, achieving an LOD of 0.2 mg L^−1^ for FA [34]. The optical fiber biosensor offered good selectivity for FA via a polyoxometalate membrane, with detection limits comparable to the reference acetylacetone method and no heating requirement. However, sensor requires sample pretreatment (protein precipitation), shows slightly lower precision than spectrophotometry, and has limited long-term stability data beyond one week.

On the other hand, earlier studies demonstrated that MEL can be detected via hydrogen bond interference in AgNP synthesis or through cysteamine-modified AgNP aggregation, resulting in distinct absorption shifts. The interference green-synthesis AgNP colorimetric biosensor enables rapid, low-cost, single-step visual detection of MEL (LOD, 0.79 µM) in the linear range of 0.79 µM to 79.3 µM, with high selectivity and HPLC validation. However, sensor requires milk pretreatment and UV–Vis confirmation for quantification and offers moderate sensitivity, limiting precise trace-level analysis and portability [35].

The colorimetric biosensor based on succinic acid-functionalized Ag NPs (Suc-Ag NPs) demonstrated a simple, low-cost, and highly selective visual approach for MEL detection in milk (0.01 µM), with good linearity (0.1–1.2 µM), satisfactory recoveries (98–111%), and RSD (0.12–0.18%) values. However, the need for milk pretreatment and limited long-term stability assessment constrain large-scale scalability [36].

Overall, colorimetric sensors offer a rapid, cost-effective, and portable solution for ensuring milk authenticity and safety. However, challenges remain in achieving simultaneous multi-analyte detection, improving scalability, and maintaining sensor stability during field applications. The integration of optical signal analysis with smartphone-based platforms and microfluidic architectures continues to push the boundaries of real-time adulteration monitoring, bridging the gap between laboratory precision and practical on-site usability [37].

#### Mechanisms, Sample Preparation, Interference Effects, Stability, and Comparison with Reference Methods in Colorimetric Sensors

Colorimetric biosensors for detecting milk adulteration function via proven optical transduction mechanisms that transform chemical or biological interactions into visible color changes. These methods often encompass nanoparticle aggregation or dispersion linked to LSPR shifts, enzyme-mediated catalytic reactions, redox-driven chromogenic responses, and pH-sensitive dye transitions [38]. In nanoparticle-based systems, interactions between adulterants and surface-modified gold or silver nanoparticles can modify interparticle spacing, leading to detectable variations in absorbance. Furthermore, enzyme-based methods generally depend on biocatalytic reactions that induce chemical interactions. Although these pathways facilitate rapid signal production, their analytical efficacy is influenced by matrix conditions and assay design [39].

Sample preparation is crucial for attaining consistent colorimetric responses, given the intricate composition of milk, which comprises proteins, lipids, lactose, and mineral salts. Several reports utilize minimum pretreatment, including dilution with water or buffer, to mitigate viscosity and matrix effects. Moreover, centrifugation, filtration, or partial fat removal has been employed to reduce light scattering and nonspecific interactions, especially in nanoparticle-based studies. Paper-based and microfluidic sensors could enhance repeatability by precisely regulating sample volume and reagent distribution [40].

Interference from intrinsic milk constituents continues to provide a significant analytical difficulty. Compounds like lactose, proteins, amino acids, salts, and preservatives may affect nanoparticle stability or enzymatic activity, potentially resulting in nonspecific responses. Selectivity has been enhanced in certain studies via surface functionalization, incorporation of biorecognition components, or multiplexed detection approaches; yet, systematic interference studies across various milk matrices are still scarce [41].

In general, biosensors exhibit significant analytical performance in controlled laboratory environments, but their practical application in milk samples is highly impeded by the intrinsic complexity of the milk matrix. The presence of high concentrations of proteins (e.g., caseins and whey proteins), lipids, lactose, minerals, and endogenous enzymes in milk samples is highly averse to sensor effectiveness. These components may nonspecifically adsorb onto sensor interfaces, clog active sites, and generate optical scattering and background fluorescence, ultimately leading to signal instability and reduced analytical accuracy. In colorimetric sensors, matrix-induced turbidity, light scattering, and inherent background coloration of milk can further obstruct visual or image-based readouts, especially at low adulterant concentrations. Consequently, this results in reduced analytical sensitivity of the colorimetric sensors.

Among these factors, protein adsorption is particularly challenging, which impedes reagent–analyte interactions and hinders mass transfer at the sensing interface. Although comprehensive systematic studies are limited, several mitigation strategies have been explored to alleviate matrix interference. These include sample pretreatment approaches such as dilution, centrifugation, and membrane filtration, which reduce protein and fat content prior to analysis. In addition, antifouling surface modifications such as polyethylene glycol, zwitterionic polymers, or hydrophilic coatings have been employed to suppress nonspecific adsorption. Ratiometric colorimetric sensing and reference-based image analysis have also emerged as effective strategies to compensate for fluctuations in illumination, sample opacity, and background interference.

In addition to matrix effects, the practical application of colorimetric sensors in real-time is further limited by concerns of stability and reusability. Biological recognition elements, such as enzymes and antibodies utilized in colorimetric assays, are prone to denaturation, leaching, and diminished activity under fluctuating pH, temperature, and storage conditions. While nanomaterials like gold nanoparticles improve optical sensitivity, they are susceptible to aggregation, oxidation, or surface remodeling over extended storage. Strategies to enhance operational robustness, such as immobilization inside polymer matrices, encapsulation in sol–gel frameworks, and the replacement of biological components with aptamers or molecularly imprinted polymers (MIPs), have been rigorously explored. Overcoming these problems is crucial for transitioning colorimetric sensors from laboratory settings to commercial applications for regular monitoring of milk adulteration [42].

The stability and shelf life of colorimetric biosensors are contingent upon their composition. For instance, inorganic nanoparticle-based sensors often have superior physicochemical stability, while enzyme- or aptamer-based devices may display diminished operating shelf life due to environmental sensitivity. The reported storage stability ranges from weeks to months, although standardized evaluation methodologies have not yet been established [43].

In comparison to reference analytical techniques, such as HPLC, GC–MS, and ELISA, colorimetric biosensors generally exhibit diminished sensitivity and specificity. Nonetheless, their affordability, ease of use, and mobility facilitate their application as initial screening instruments, with validation conducted by recognized laboratory techniques [44].

### 2.2. Fluorometric Sensors

Fluorometric sensors have emerged as highly sensitive, selective, and rapid analytical tools for detecting adulterants in milk and other food products. These sensors rely on fluorescence-based mechanisms such as FRET, ratiometric fluorescence, and QD-based detection, enabling accurate and portable detection of adulterants in complex matrices. Hafez et al. designed a FRET-based fluorometric sensor utilizing AuNBPs and UCNPs [9]. The tunable LSPR of AuNBPs acted as a dynamic quencher for UCNPs, providing a highly sensitive and ratiometric sensing platform for urea detection with a low LOD of 0.056 μM. The biosensor exhibits exceptional analytical sensitivity, with submicromolar detection limits achieved through ratiometric fluorometric and colorimetric readouts. The dual-mode strategy, ratiometric FRET mechanism, and smartphone-assisted analysis effectively reduce background interference and enhance quantitative reliability in milk matrices. The validation against a commercial urea assay confirms the analytical precision. However, the system relies on enzymatic hydrolysis, multistep sample pretreatment, and multiple reagents, increasing operational complexity. Fabrication involves sophisticated nanomaterial synthesis, which may limit scalability, cost efficiency, and widespread field employment despite strong laboratory performance.

Similarly, Zhang et al. reported a perylene derivative probe-based fluorescence sensor array capable of distinguishing various concentrations of milk adulterants with excellent linearity and without requiring bulky instruments, making it cost-effective and suitable for routine analysis [45]. In recent studies, fluorescence-based sensors have been used for identifying adulterants in other food products. Fluorescence spectroscopy has also been explored for identifying edible oil adulteration in milk. The sensor array exhibits significant strengths, including non-targeted detection capability, high classification accuracy (100% cross-validation), rapid response (<10 s), and the ability to discriminate multiple adulteration scenarios, such as plant protein and MEL addition. More importantly, the array-based approach avoids the need for highly specific receptors and demonstrates strong pattern recognition performance. However, the system relies on multichannel fluorescence measurements, statistical linear discriminant analysis (LDA), and sample dilution, which limits straightforward point-of-use deployment. Additionally, the method is indirect and semi-quantitative, with performance dependent on data processing rather than absolute analyte concentration, and long-term stability and portability are not fully addressed.

Choudhary et al. developed CQD-based fluorometric sensors for urea detection, utilizing a commercial optical fiber spectrometer (OFS) and color sensor device (CSD) to track fluorescence changes induced by urea hydrolysis [46]. The resulting fluorescence sensor demonstrated notable strengths, including high photostability, good linearity, and accurate detection of milk spoilage and urea adulteration within relevant concentration ranges. The embedded optical setup and low-cost electronics enhance portability and support near real-time analysis, with validation against a standard pH meter showing strong agreement. However, the sensing strategy is indirect, relying on pH changes induced by urea hydrolysis, which may limit selectivity in complex matrices. Additionally, enzymatic dependence, multi-step sample preparation, and limited long-term storage stability data constrain large-scale commercialization.

In a similar study, Alanazi et al. designed a ratiometric fluorometric sensor using copper (II)-quenched near-infrared-emitting CDs and methyl red-quenched red-emitting CDs (R-CDs/MR and NIR-CDs/Cu^2+^), demonstrating strong pH-dependent fluorescence changes and remarkable resistance to environmental interference [47]. The urease-assisted ratiometric fluorescence biosensor exhibited excellent sensitivity (LOD, 0.28 µM), wide linear range, and strong resistance to matrix interference, benefiting from built-in ratiometric self-calibration. The sensor showed good recoveries in milk and robust photostability, thermal stability, and ionic tolerance. However, its performance relies on enzymatic hydrolysis, controlled incubation (30 min at 37 °C), and protein precipitation pretreatment, limiting rapid field deployment. The requirement for dual fluorophores and spectrofluorimetric readout may also constrain cost-effective miniaturization and industrialization.

Formaldehyde, a common milk adulterant, has been targeted by several fluorometric systems due to its carcinogenicity [48]. Du et al. developed a naphthalimide-based fluorometric (NFD) sensor on filter paper for FA detection across various foods, achieving recoveries between 82.1% and 111.5% [49]. The sensor demonstrated notable strengths, including high selectivity for FA, low micromolar detection limits, good reproducibility (RSD < 4%), and successful validation in diverse real food and animal serum samples. The probe also enables dual liquid-phase and solid-state (paper-based) detection, supporting visual and semi-portable analysis. However, the sensing strategy requires relatively long reaction times (~40 min), organic co-solvents (50% DMSO), and sample extraction steps, limiting point-of-care use. Sensitivity remains inferior to electrochemical approaches, and long-term storage stability, reusability, and large-scale manufacturing robustness are not comprehensively assessed, which may constrain translational applicability.

Chen et al. fabricated a dual-mode FA sensor combining a model molecule (NAHN) with InP/ZnS QDs, achieving fluorescence and colorimetric detection with limits of 0.623 μM and 0.791 μM, respectively [50,51]. The biosensor demonstrated several notable strengths, including a simple preparation strategy based on mechanical mixing, operation in neutral aqueous media, and clear visual readouts enabled by ratiometric fluorescence and color change. The use of InP/ZnS QDs as an internal reference improved signal reliability and reduced false positives, while smartphone-assisted visualization and film/glove formats supported portability and on-site use. The sensor exhibited good selectivity toward FA and satisfactory recoveries (90.50–109.17%), with RSDs of 2.12–6.38% in real water samples. However, limitations include a moderate response time (up to ~30 min), micromolar-level detection limits that may not capture trace contamination, and limited discussion on long-term storage stability and batch-to-batch reproducibility, which may hinder large-scale applications.

Melamine, another toxic nitrogenous adulterant, has been widely investigated using fluorometric approaches. Dhenadhayalan et al. constructed a thiol-functionalized molybdenum diselenide (MoSe_2_/SH QDs) fluorescent probe that relies on metalion-induced activation and energy transfer inhibition [52]. The biosensor demonstrated notable strengths, including high fluorescence stability, tunable surface chemistry, and versatile multisensing capability, enabling selective detection of Cu^2+^, 2,4,6-trinitrophenol (TNP), and MEL through distinct mechanisms such as fluorescence turn-on, electron transfer, and FRET suppression. The sensor exhibited low nanomolar detection limits (27.7 nM), good photostability (≥4 months), and satisfactory recoveries in real water and milk samples. However, the platform requires buffered conditions (pH 7.4), controlled incubation, and fluorescence instrumentation, limiting point-of-care applications. Furthermore, the approach remains single-analyte per functionalization, and scalability, cost-effective manufacturing, and regulatory validation were not comprehensively addressed.

Taşci et al. created a rapid polymeric fluorometric sensor using MEL-selective acrylate citric acid (ACA) with a response time of one minute [53]. The sensor exhibited several strengths, including high noteworthy selectivity toward MEL, a low detection limit (0.232 nM), rapid response (<1 min), and excellent reusability (≥250 cycles), which significantly enhances cost-effectiveness. Validation against HPLC confirms analytical accuracy, supporting its reliability for milk powder analysis. However, the method requires chemical pretreatment using trichloroacetic acid (TCAA), controlled pH conditions (pH 6.0), and fluorescence instrumentation, which limits portability. In addition, short- and mid-term stability were demonstrated, where scalability and real-time on-site integration remained insufficiently addressed.

The terbium-doped graphene QD (Tb-GQD) fluorescence biosensor exhibited convenient advantages, including simple microwave-assisted synthesis, high photostability, dual-emission behavior, and satisfactory sensitivity toward MEL (LOD ≈ 0.31 µM) [54]. The probe also showed good selectivity, acceptable recoveries (100.25% to 115.4%) in spiked milk samples, and a clear static-quenching mechanism supported by lifetime analysis. The sensing protocol requires chemical pretreatment (i.e., TCAA), pH adjustment, and fluorescence spectroscopy, limiting direct on-site applicability. Detection limits remained in the micromolar range, which may be insufficient for ultra-trace monitoring, and long-term storage stability, batch-to-batch reproducibility, and device-level integration were not comprehensively evaluated, thereby constrained for large-scale applications.

The PEI-stabilized AgNP fluorescent biosensor demonstrated a simple and green synthesis route, visible fluorescence response, and good selectivity toward MEL via hydrogen bonding and Ag^+^ coordination mechanisms [55]. The sensor achieved a moderate detection limit (132 nM) with a wide linear range (0.16–56 µM) and satisfactory recoveries (100.1–107.3%) in spiked milk samples, indicating feasibility of sensors. However, limitations remain, including reliance on sample pretreatment to remove proteins and fats, moderate sensitivity compared with recent nanocluster or aptamer-based platforms, and limited long-term shelf-life evaluation. Moreover, validation against reference chromatographic methods and scalability were not comprehensively addressed.

Zhuang and his coworkers developed a turn-on fluorescence biosensor based on nitrogen-doped carbon dot (N-CQDs)–Fe^3+^, which offered beneficial features such as simple one-step synthesis, high fluorescence quantum yield (~51%), good aqueous stability (up to 120 days), and acceptable selectivity toward MEL via competitive Fe^3+^ coordination [56]. The sensor demonstrated satisfactory recoveries (92.5–105.4%) in spiked milk samples, signifying practical feasibility. However, its detection limit (0.66 µM) is not relatively high compared with more recent nanomaterial-based sensors, and the response requires ~30 min incubation, limiting rapid screening. In addition, reliance on Fe^3+^ mediation introduces susceptibility to metal ion interference under complex matrices, and portability and device integration were not addressed.

In another study, Xie et al., designed turn-on fluorescent biosensor using DNA–Ag nanocluster (DNA-AgNC), which offers label-free operation, simple synthesis, rapid response (~15 min), and a detection limit of 0.1 μM, which is well below the regulatory safety threshold for MEL in dairy products [57]. The sensor demonstrates acceptable selectivity, satisfactory recoveries (98.75–101.25%), and RSD (4.8–5.9%) values in real milk samples following pretreatment. However, its performance relies on Hg^2+^-mediated fluorescence quenching and recovery, introducing potential toxicity and interference concerns. The requirement for acid extraction, centrifugation, and filtration limits POC applications. Furthermore, sensitivity remains moderate compared with recently designed nanomaterial- or electrochemical-based sensors, and long-term stability, portability, and scalability were also not addressed.

The conjugated polyelectrolyte-stabilized AgNP (P1–AgNPs) ratiometric biosensor was developed by Zhu and his colleagues [58]. The sensor exhibited ultrasensitive MEL detection (LOD, 0.1 nM for fluorometric; 0.45 nM for colorimetric), dual-signal self-calibration, and naked-eye readability, which enhance analytical reliability in complex milk matrices. The aggregation-induced ratiometric mechanism improved sensing precision compared with single-signal sensors and achieved good recoveries (≈99–114%), which demonstrated applicability to real milk samples. However, the sensor requires alkaline pH (≈9), incubation (~15 min), and acid-based sample pretreatment, limiting their application in industrial applications. In addition, long-term storage stability, batch reproducibility, and scalable device integration were also not addressed, constraining their further applicability.

Collectively, fluorometric sensors offer remarkable advantages, including high sensitivity, real-time response, and the potential for miniaturization into portable formats for on-site milk adulteration detection (Table 2).

#### Mechanisms, Sample Preparation, Interference Effects, Stability, and Comparison with Reference Methods in Fluorometric Sensors

Fluorometric biosensors for detecting milk adulteration utilize fluorescence transduction processes to transform analyte–probe interactions into quantifiable emission variations. Typical principles include FRET, ratiometric fluorescence, fluorescence quenching or enhancement, and QD-mediated energy transfer. For instance, FRET-based systems utilize distance-dependent energy transfer between donor and acceptor entities, while ratiometric sensors employ dual-emission signals to enhance measurement reliability. These techniques provide elevated sensitivity but necessitate regulated optical conditions to ensure consistency [59].

Sample preparation is a significant aspect due to the turbidity and intricate composition of milk, which comprises proteins, lipids, and dissolved particles that may influence fluorescence measurements. Many fluorometric sensors utilize straightforward dilution or buffer modification to mitigate scattering and inner-filter effects. In addition, centrifugation, filtering, or enzymatic pretreatment is utilized to reduce matrix influence. Paper substrates and fiber-optic arrangements provide regulated sample management and enable portable analysis [60].

Interference from intrinsic milk components poses a significant analytical difficulty. Different proteins, lactose, metal ions, preservatives, and pH fluctuations may affect fluorescence intensity, quenching efficiency, or probe stability. Selectivity is frequently enhanced via the surface functionalization of nanomaterials, the integration of biorecognition components, and ratiometric signal correction; however, comprehensive interference studies across various milk matrices are still scarce [61].

Fluorimetric biosensors have emerged as effective analytical instruments for detecting milk adulterants due to their high sensitivity, minimal detection limits, and appropriateness for trace-level investigation. However, their application in actual milk samples is considerably limited by the inherent complexity of the milk matrix, which can directly interact with fluorescent probes, leading to adverse fluctuations in the fluorescence outcomes. These substances may adsorb onto probe surfaces and interfere with energy transfer processes, thereby inhibiting fluorescence intensity and affecting long-term stability.

Matrix-induced interference is especially significant in fluorescence-based systems. The turbidity of milk diminishes excitation and emission light, while naturally existing fluorophores such as riboflavin, tryptophan residues, and vitamins generate background autofluorescence that interferes with sensor emission bands. These effects can mask fluorescence responses caused by analytes by FRET, PET, or the IFE. Additionally, both static and dynamic quenching mechanisms may arise from complex formation or collisional interactions with milk proteins, metal ions, or dissolved oxygen, hence affecting signal interpretation and calibration precision. Despite the limited scope of comprehensive mechanistic investigations, various mitigation strategies have been examined, such as dilution, centrifugation, and fat removal pretreatments, in addition to spectral filtering, background subtraction, lifetime-based detection, and ratiometric fluorescence techniques to counteract matrix-derived interference.

Numerous fluorescent probes, such as organic dyes and semiconductor nanomaterials, are prone to photobleaching, chemical degradation, and signal drift. For example, fluorescent nanomaterials, including metal nanoclusters and carbon dots, may experience oxidation, aggregation, and loss of physicochemical properties during storage, leading to diminished quantum yield. To improve robustness, immobilization within polymer matrices, silica encapsulation, and integration into hydrogel networks have been extensively studied. Recently, aptamers and MIPs have demonstrated enhanced stability and reusability to environmental variations in many fluorimetric sensors [62].

The stability and shelf life of fluorometric sensors are contingent upon the composition of the probe. For instance, QD-based probes typically demonstrate excellent photostability, but organic fluorophores and enzyme-assisted systems may experience photobleaching or a decline in activity. Additionally, the shelf life of the sensors often varies from a few weeks to a few months, contingent upon storage conditions.

Fluorometric biosensors, unlike traditional analytical methods such as ELISA, HPLC, and GC–MS, demonstrate diminished analytical precision while offering advantages in response time, portability, and ease of use, making them advantageous for on-site milk quality assessment [63].

### 2.3. Electrochemical Sensors

Electrochemical sensors have emerged as indispensable analytical tools for detecting adulterants in milk due to their high sensitivity, rapid response, and cost-effectiveness. The key electrochemical parameters, such as impedance, phase angle, charge transfer resistance, and electrical conductivity, are primarily responsible for the precise determination of adulterants in milk. For instance, Yadav et al. demonstrated that the impedance magnitude increases with the concentration of FA adulteration, exhibiting a strong linear correlation with adulterant levels [64]. The sensor offered several advantages, such as extremely simple fabrication, low material cost, minimal sample volume (≈4 mL), good repeatability, and label-free detection of FA in milk. The identification of optimal frequency windows (40–200 kHz for impedance and 100–300 Hz for phase angle) enhanced measurement robustness. However, the sensor exhibited a relatively high detection limit (≈1–2.5% *v*/*v*), restricting sensitivity to gross adulteration rather than trace-level monitoring. Moreover, the approach is non-selective, responding to bulk conductivity changes, and potential interferences from other ionic adulterants exist, which limited sensor’s specificity.

Similarly, Tripathy et al. developed microfabricated Au electrodes on glass substrates and compared their sensitivity to that of Pt electrodes on GCE [65]. Their study revealed comparable efficiency, leading to the development of a cost-effective lab-on-chip platform for multiplexed analysis of various milk adulterants based on fluctuations in electrical impedance and pH. The impedance–pH-based sensor offered low cost, simple fabrication, and broad sensing capabilities for multiple milk adulterants without specific receptors. The higher detection limits, indirect selectivity, and sensitivity to natural milk variability, limiting trace-level detection and lacking an extensive validation against reference methods.

Complementary research has also reported the successful use of electrochemical impedance spectroscopy (EIS) for efficient determination of multiple adulterants in milk [66,67,68]. Enzymatic sensors have been widely utilized in the electrochemical detection of milk adulterants, particularly urea, owing to their specificity and catalytic properties. A range of urease (Urs)-based biosensors have been reported, including Fe_3_O_4_/MWCNT/PANI-Nafion/Urs/GCE, graphene nanoplatelet and graphitized nanodiamond composites (functionalized-GNPlts/GNDs/Urs/SPE), AuNPs/GO/Urs/GCE, and Nf/PANI/CuF/Urs/GCE configurations [69,70,71,72]. Among these, the Nf/PANI/CuF/Urs/GCE composite exhibited enhanced electrochemical performance attributed to the lower bandgap values of copper ferrite (CuF) nanoparticles, which facilitated superior electron transfer kinetics [71]. The Nf/PANI/CuFe_2_O_4_/urease electrochemical biosensor demonstrated low detection limits (0.17 μM), wide linear range (0.5–45 μM), good selectivity, and successful validation in milk samples, highlighting its analytical relevance for food safety monitoring. The use of CuFe_2_O_4_ NPs enhanced electron transfer due to their narrow bandgap, resulting in improved sensitivity compared with other ferrites. However, the biosensor remained enzyme-dependent, which limited long-term operational stability, with measurable signal degradation beyond one week. Additionally, the multi-step fabrication and surface functionalization may pose challenges for large-scale manufacturing and batch-to-batch reproducibility.

The incorporation of metal-based nanomaterials such as AuNPs further enhanced conductivity and surface area, promoting efficient electron transport and improving the electrochemical response of the biosensor [72]. The GCE/AuNPs/GO/watermelon-urease biosensor demonstrated several strengths, including high sensitivity (LOD ≈ 6.16 μM), wide linear range (0.83–15.0 mM), excellent selectivity (ksel < 0.1), and direct analysis of raw milk without pretreatment. The use of plant-derived urease offered cost effectiveness, sustainability, and improved catalytic activity compared with commercial enzymes. Importantly, the sensor exhibited good long-term stability (~4 months) and batch-to-batch reproducibility, with validation against the DMAB reference method. Moreover, the platform remains enzyme-dependent, which may limit robustness under harsh conditions, and the need of multi-step fabrication may constrain large-scale and POC applications.

However, the practical use of enzymatic sensors remains limited due to inherent challenges such as enzyme immobilization instability and fluctuations in enzyme activity under varying environmental conditions, such as pH, temperature, and humidity [73]. These limitations have driven the transition toward non-enzymatic electrochemical sensors, which offer higher stability, reusability, and operational simplicity.

In this context, Sha et al. developed a graphene–polyaniline (Gr–PANI)-based electrochemical sensor for urea detection, which exhibited superior performance, over four times higher than that of pure PANI-based sensors and sixty times greater than pure graphene sensors [74]. The sensor’s sensitivity was significantly improved by tuning the PANI layer thickness, achieving a low LOD of 5.88 μM within a wide linear range of 10–200 μM. This biosensor delivered many advantages such as enzyme-free operation, simple electro-deposition fabrication, good reproducibility (RSD < 6%), and stable performance over repeated measurements. The synergistic interaction between graphene and PANI significantly enhanced electron transfer, yielding improved sensitivity compared with single-component electrodes. The sensor also showed acceptable selectivity against common interferents and successful validation in milk samples. However, the detection limit remains in a moderate micromolar range, limiting applicability for trace-level adulteration monitoring. Additionally, the reliance on controlled buffer conditions and lack of device-level integration or long-term shelf-life evaluation may restrict translation to portable and on-site dairy testing platforms.

Similarly, Khataee et al. designed an electrochemical sensor utilizing a La-doped CoFe layered double hydroxide on reduced graphene oxide (La–CoFe LDH@rGO) nanocomposite, where synergistic interactions between LDH and rGO enhanced electrocatalytic activity [75]. This sensor achieved an impressive LOD of 0.33 ± 0.11 μM within a broad concentration range (0.001–23.5 mM) and exhibited a swift response time (5 s) at a low working potential (0.4 V). Such low-potential electrochemical responses are particularly advantageous for minimizing interference from common electroactive interferents like ascorbic acid (AA) and uric acid (UA). The enzyme-free operation, high electrocatalytic activity, rapid response (~5 s), a wide linear range, and a low detection limit are the key merits of this sensor. The synergistic integration of La-doped LDH with rGO enhanced electron transfer, surface area, and long-term stability, with ~90% signal retention over three months and good reproducibility (RSD < 3%). Validation in milk and serum matrices demonstrates practical applicability. However, operation in strongly alkaline media, partial sensitivity to glucose interference, and limited evaluation under highly complex matrices may constrain for real sample analysis.

In another study, Magar et al. designed an electrochemical impedimetric sensor for urea detection using a CuO/Co_3_O_4_@MWCNTs/SPE nanocomposite [76]. This configuration displayed ultrasensitive picomolar-level sensing with an exceptionally low LOD of 0.223 pM and a wide linear range of 1.0 pM–10 mM. The sensor’s excellent anti-interference ability and stable performance at a low working potential (0.7 V) highlighted its potential for field-deployable milk analysis. The biosensor exhibited exceptional analytical performance, including an ultra-low detection limit, very wide linear range, rapid response, and enzyme-free operation, which collectively enhanced stability and shelf life. The use of screen-printed electrodes (SPEs) supported low-cost fabrication and portability, while good repeatability and selectivity against common interferents were also demonstrated. However, the sensor was operated under strongly alkaline conditions (0.1 M KOH) and relies on EIS analysis, which may limit POC applications. Additionally, large-scale manufacturing, long-term storage beyond weeks, and regulatory validation against standardized reference methods remain insufficiently addressed.

Similar non-enzymatic sensors developed by Naik et al. have also demonstrated stable electrochemical performance at comparable working potentials [77].

Over the decades, NO_2_^−^ and SO_3_^2−^ have been extensively used as food additives and preservatives in the food industry. However, excessive consumption of NO_2_^−^ has been linked to severe health complications, including methemoglobinemia (blue baby syndrome), gastrointestinal distress, and an increased risk of stomach cancer, while SO_3_^2−^ may induce allergic reactions and oxidative stress. Despite their classification as generally recognized as safe (GRAS) by the U.S. FDA, global overuse remains a major concern [78,79].

Under European Union (EU) food safety legislation, the intentional addition of NO_2_^−^ and (SO_3_^2−^, expressed as SO_2_ equivalents) is not permitted in milk, and their presence is regarded as an adulteration. However, their addition in some foods is allowed, which include fish, meats, and cheese, including sodium and potassium salts of nitrite. The daily intake limits for NO_3_^−^ and NO_2_^−^ in these foods include 3.7 and 0.07 mg/kg/body weight per day. Consequently, SO_3_^2−^ must be declared when present at concentrations ≥ 10 mg kg^−1^ (as SO_2_) in some foods and milk, necessitating sensor-based detection approaches for consumer protection [80].

Given their potential toxicity, numerous electrochemical sensors have been developed for the quantification of these compounds. Although sensors for NO_2_^−^ detection in food products such as sausages, pickles, and meat have been well-studied, their application in milk matrices remains relatively limited [81,82]. Zhang et al. synthesized acid-treated Fe_3_O_4_@SiO_2_ NPs for detecting NO_2_^−^ and SO_3_^2−^ through induced electrostatic interactions, achieving micromolar-level sensitivity [83]. As-designed sensor offered simple fabrication, good selectivity, and acceptable sensitivity for NO_2_^−^ (LOD, 3.33 μM) and SO_3_^2−^ (LOD, 31.57 μM) within regulatory ranges. However, its micromolar detection limits, reliance on benchtop electrochemical instrumentation, and lack of long-term shelf-life assessment may restrict POC use in dairy monitoring.

To overcome these limitations, Arivazhagan et al. fabricated highly sensitive Au nanodendrite (Au ND)-based flexible screen-printed carbon electrodes (FSPCEs) for NO_2_^−^ detection in milk [84]. The Au NDs@FSPCEs acted as rapid and efficient electrocatalysts, providing an exceptional sensitivity of 52.529 μA μM^−1^ cm^−2^ and an LOD of 1.0 nM. These results were attributed to the significantly increased electrochemically active surface area (ECSA) and superior electrical conductivity of the Au nanodendrites, demonstrating their potential in flexible sensing applications. The sensor exhibited outstanding analytical performance with a lowered detection limit, wide linear range, rapid response (<3 s), high sensitivity, and selectivity toward NO_2_^−^ sensing in milk samples. The one-step, binder-free electrodeposition, and mechanical flexibility supported scalability and POC use, with good reproducibility, satisfactory recoveries (97.49–99.47%), and good RSD (1.19–3.44%) values in milk samples. However, the sensor required operation at relatively high anodic potentials, which may increase susceptibility to fouling over prolonged use. Furthermore, long-term storage stability, batch-to-batch variability of nanodendrite morphology, and robustness under repeated use remained only partially addressed, requiring further validation for commercialization.

**Figure 2:**
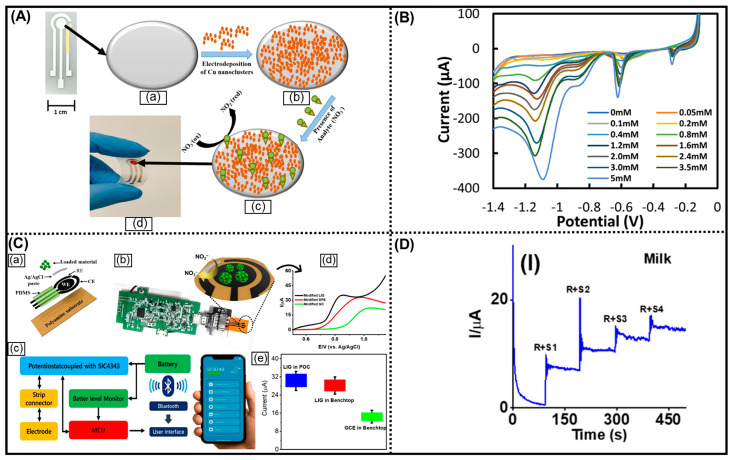
Smart analytical strategies for the determination of nitrates and nitrites. (**A**) Schematic illustration of the fabrication process and the detection mechanism of the NO_3_^–^ sensor. (**a**) Screen-printed silver (Ag) WE (**b**) modified with electrodeposited copper (Cu), leading to (**c**) NO_3_^–^ reduction. (**d**) Micrograph of the cost-effective, flexible, screen-printed electrochemical NO_3_^–^ sensor. (Reproduced from Ref. [85]). (**B**) LSV at different concentrations of NO_3_^–^ in 0.1 M KCl. (Reproduced from Ref. [85]). (**C**) (**a**) Illustration of a flexible MXene/NiCoMn-LDH/S-LIG sensor for on-site detection, (**b**) smartphone-based electrochemical system, (**c**) detector architecture, (**d**) NO^2–^ sensing via CV oxidation peaks on LIG electrodes, and (**e**) comparison of peak currents using benchtop and portable potentiostats (Reproduced from Ref. [86]). (**D**) Chronoamperometry performance of the sensor for NO^2−^ detection in milk sample. (Reproduced from Ref. [86]).

In a similar study, Inam and his coworkers designed a flexible screen-printed amperometric electrochemical nitrate (NO_3_^−^) sensor functionalized with electrodeposited copper metal nanoclusters. By integrating both screen printing and electrodeposition techniques, the sensor is highly cost-effective and scalable. The sensor also demonstrated a significant ability to detect NO_3_^−^ in water, exhibiting a low LOD of 0.207 nM from concentrations between 50 and 5000 μM via linear sweep voltammetry (LSV) (Figure 2A,B) [85]. Recently, smartphone-based POC systems have attracted researchers’ interest globally. In this regard, Ranjith et al. designed a portable and flexible MXene/NiCoMn-LDH/S-laser-induced graphene (LIG) hybrid electrode and a portable detector, integrated with an Android application installed on a smartphone, to assess the electrochemical performance for NO_2_^−^ detection in a POC system (Figure 2C,D). Notably, the signal from LIG-based electrodes surpasses that of conventional GCE-based electrodes, leading to heightened sensitivity of the LIG surface, which are attributed to their higher electrical conductivity and EASA [86]. The biosensor showed high sensitivity, low detection limit, wide linear range, strong anti-interference capability, and excellent stability features via its hierarchical heterostructure and smartphone-integrated portability. However, material synthesis is relatively complex, and long-term robustness under diverse conditions requires further validation.

**Figure 3:**
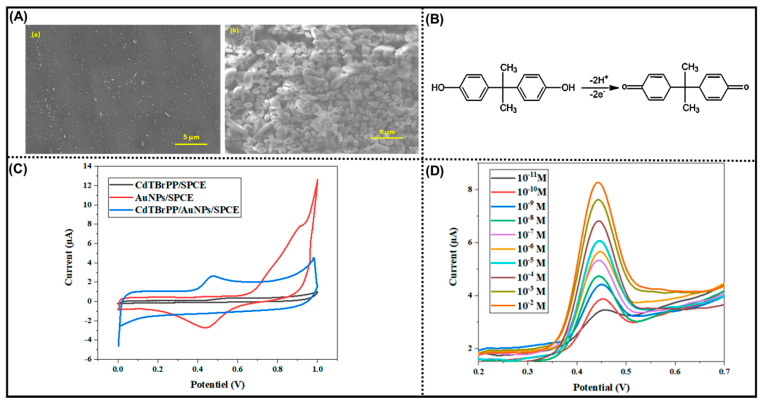
Flexible electrochemical sensor for BPA detection. (**A**) SEM images of the modified electrode: (**a**) AuNPs film and (**b**) AuNPs/CdTBrPP film. (**B**) Electrooxidation mechanism of BPA. (**C**) Electrochemical behavior of AuNPs/SPCE, CdTBrPP/SPCE, and AuNPs/CdTBrPP/SPCE in PBS with 1 µM BPA. (**D**) SWV response of CdTBrPP/AuNPs/SPCE at various concentrations of BPA in PBS. (Reproduced from Ref. [91]).

Another widespread adulterant of concern is bisphenol A (BPA), a monomer commonly used in the manufacturing of food packaging materials. Bisphenol A leaching from containers into food and beverages has been associated with endocrine disruption, reproductive toxicity, and neurodevelopmental disorders [87]. Due to its severe health impacts, BPA usage has been banned in several countries. The European Food Safety Authority (EFSA) further reduced the tolerable daily intake (TDI) level for BPA from 4 µg/kg to 0.2 ng/kg body weight/day [88,89]. Owing to these safety concerns, electrochemical sensors have gained traction for sensitive BPA detection. Ma et al. developed an AuNPs/boron-doped diamond (BDD)-based aptasensor with excellent linearity from 1.0 nM to 10 fM and an ultralow LOD of 7.2 fM [90]. The sensor demonstrated high stability and reproducibility in milk samples with optimal recovery values. The ultrasensitive sensor is highly demanding to achieve for BPA detection among a wide variety of sensors. For instance, Rejab et al. developed a novel electrochemical sensor using a chemical compound, i.e., metalloporphyrin, which offered exceptional stability, low toxicity, easy production, and efficient electron transport. The metalloporphyrin provides a robust π-conjugated electron system and a core metal, which function as sensitizers and electron mediators. The morphology of the as-prepared working electrode, i.e., AuNPs/CdTBrPP, is desirable with the smooth and uniformly textured film-like appearance of AuNPs and the aggregated immobilized CdTBrPP layer (Figure 3A). The strong affinity between BPA and the CdTBrPP is attributable to the specific coordination of the central cadmium ions with the hydroxyl group of BPAs, while π–π interactions between the porphyrin ring and the aromatic structure of BPA may augment selectivity (Figure 3B). The higher oxidation peak intensity observed in the CdTBrPP/AuNPs/SPCE was attributed to the efficient charge transfer from AuNPs (Figure 3C). The sensor showed a better electrochemical response towards BPA in the concentration range of 10^−11^ to 10^−2^ M, with a picomolar detection limit around 9.5 pM and a high sensitivity of 0.52 (Figure 3D) [91]. Ensafi et al. further enhanced BPA sensing by constructing a molecular imprinted polymer (MIP)-based aptamer sensor integrating polypyrrole (Ppy), DNA sequence (p-63), and AuNPs (PPY/@p-63/AuNPs/GCE) [92]. This configuration achieved femtomolar-level detection, exhibiting a wide linear range (0.5 fM–5.0 pM) and an ultralow LOD of 80 aM, confirming its superior analytical performance in milk matrices. The aptamer–MIP electrochemical biosensor offered an ultrasensitive attomolar detection, excellent selectivity, and good short-term stability demonstrating strong analytical capability. However, complex multi-step fabrication, costly aptamer components, and reliance on EIS instrumentation limit scalability, portability, and routine operation despite outstanding sensitivity.

Despite their exceptional sensitivity, many of these advanced sensors require sophisticated fabrication and skilled personnel, limiting their scalability and affordability. Addressing these challenges, Dey et al. developed a pyridine-2,6-dicarboxylic acid-based metal–organic framework (Ni–Cu(PDA)MOF) integrated with carbon nanofiber (CNF) paper [93]. The resulting Ni–Cu(PDA)MOF/CNF/GCE sensor exhibited enhanced porosity, hydrophilicity, and extensive surface area, facilitating efficient redox reactions with a detection limit of 75 nM. Notably, the sensor operated under neutral conditions (pH 7.0) with irreversible diffusion-controlled oxidation, offering practical feasibility for BPA monitoring. The sensor demonstrated high sensitivity, wide linear range, strong anti-interference capability, and good one-month storage stability, validated against HPLC. However, multi-step fabrication, reliance on laboratory electrochemical instrumentation, and limited portability may hinder industrial use in dairy monitoring.

Other recent studies have also demonstrated reliable electrochemical performance for BPA detection in adulterated milk samples [94,95,96].

Melamine contamination has posed another major food safety issue, especially in dairy products and infant formulas. Owing to its high nitrogen content, MEL is illicitly added to artificially inflate apparent protein levels. To tackle this challenge, researchers have developed various electrochemical strategies. Dong et al. constructed an MEL sensor by tuning interfacial Schottky barriers in heterojunction-based materials, which significantly enhanced sensitivity [97]. He et al. further advanced this approach by fabricating a carbon cloth-based carbon nanopoint mesh (CC/CeO_2_/CNPs) sensor [98]. Through photo-induced modulation of the Schottky barrier, this device achieved strong resistance against interference and enhanced signal intensity, yielding a fivefold sensitivity increase with an LOD of 0.274 nM in milk powder samples. This highlights the promising potential of Schottky barrier engineering in next-generation MEL sensors. The sensor delivered high sensitivity (sub-nanomolar LOD), strong anti-interference capability, and good agreement with GC–MS, enabled by Schottky-barrier modulation. However, reliance on external light sources, multi-step electrode fabrication, and specialized electrochemical instrumentation may limit portability and scalability.

In a commercial context, Algethami et al. designed a transition metal oxide-based CuO–CNT nanocomposite sensor (CuO–CNT NCs/Nafion/GCE) for MEL detection [99]. This platform offered rapid response (10 s) and excellent sensitivity (LOD 0.27 nM), validated across multiple commercial milk brands. The device’s robust performance and affordability make it suitable for large-scale food safety monitoring. The biosensor demonstrated sub-nanomolar detection, fast response, good selectivity, and successful validation in milk samples. However, multi-step nanocomposite synthesis, reliance on Nafion-modified GCEs, and limited long-term stability data may constrain large-scale manufacturing.

Similarly, Gu et al. fabricated a label-free impedimetric immunosensor using a poly(pyrrole-co-pyrrole-1-propionic acid) and rGO polymer layer for MEL detection [100]. The pyrrole-based copolymer significantly improved electron transfers due to abundant carboxyl groups and enhanced antibody binding sites. This sensor achieved high sensitivity across a broad range (10 pM–10 mM), with an LOD of 12 pM and recovery rates between 84.6% and 100.3% in various dairy matrices such as whole milk, infant formula, and ice cream. The immunosensor displayed ultrasensitive detection limits, wide linear range, good reproducibility, and successful validation against HPLC–MS method. However, antibody dependence, prolonged incubation time (150 min), and multistep sample pretreatment may limit portability, throughput, and cost-effective field deployment.

Further innovation was demonstrated by Brazys et al., who constructed an MIP-based electrochemical sensor by decorating AuNPs onto a Ppy composite for MEL sensing [101]. The MIP–PPy–AuNPs sensor exhibited excellent linearity from 50 nM to 5 μM with a detection limit of 0.83 nM. Its validation across multiple milk and infant formula samples confirmed its practical applicability in real-world dairy analysis. Overall, these studies signify the rapid advancement of electrochemical sensing platforms for milk adulteration detection (Table 3). The incorporation of nanostructured materials, heterojunction modulation, and MIP-based selectivity has enabled ultrasensitive and reliable detection of diverse adulterants, including urea, NO_2_^−^, BPA, and MEL. Future developments should focus on integrating these electrochemical sensors into portable and wearable analytical systems to facilitate continuous, on-site monitoring of milk safety and quality in real time.

#### Mechanisms, Sample Preparation, Interference Effects, Stability, and Comparison with Reference Methods in Electrochemical Sensors

Electrochemical sensors operate by transforming analyte-induced alterations at the electrode–electrolyte interface into quantifiable electrical signals. Mostly, mechanisms rely on fluctuations in impedance, charge transfer resistance, redox current, and electrical conductivity, typically assessed by EIS, amperometry, and voltammetric methods. Non-enzymatic sensors deliver concentration-dependent responses through direct electrocatalytic oxidation or reduction in target analytes at charged electrode surfaces, while enzymatic sensors depend on biocatalytic activities that generate electroactive species. The electrochemical performance of sensors is significantly affected by the electrode material, surface modification, and the applied potential [26].

Meanwhile, sample preparation is crucial because of the intricate matrix of milk that might influence electrochemical responses. Most research utilizes basic dilution using electrolyte or buffer solutions to diminish viscosity and matrix effects. Significantly, electrode fouling can be reduced using centrifugation and filtration processes, thereby enhancing signal repeatability. Furthermore, the utilization of screen-printed and microfabricated electrodes enhances controlled sample manipulation and enables portable measurements [102].

Interference from concurrent electroactive species compromises the sensitivity and specificity of electrochemical sensors. Common interferants, such as AA, UA, carbohydrates, proteins, preservatives, and a few inorganic ions, induce nonspecific signals, leading to fouling. Selectivity is frequently enhanced via nanomaterial-based surface modification, low-potential operation, and differential measurement techniques; nonetheless, comprehensive interference evaluations in milk are still scarce [103].

Recently, a myriad of electrochemical biosensors has been thoroughly investigated for the detection of milk adulterants owing to their elevated sensitivity, swift signal transduction, minimal power consumption, and compatibility with portable devices. Nonetheless, their utilization in real milk samples is constrained by the intricate physicochemical properties of the milk matrix. Milk has proteins, lipids, minerals, and endogenous electroactive molecules that can engage with electrode surfaces, leading to signal distortion and diminished analytical precision. The interferents may nonspecifically adsorb onto electrodes, obstruct catalytic sites, and hinder the transport of mass.

Electrode fouling is a significant challenge in electrochemical sensing. The adsorption of caseins, whey proteins, and fat globules creates insulating layers that inhibit electron-transfer kinetics and modify interfacial characteristics. This phenomenon is especially concerning in voltammetric methods like CV, DPV, and SWV, where surface accessibility significantly affects peak current and peak potential. In amperometric sensors, fouling can result in unsteady steady-state currents and diminished signal repeatability. Moreover, fluctuations in ionic strength and the presence of naturally occurring redox-active chemicals in milk may produce background currents that interfere with analyte signals, complicating quantitative analysis. Electrochemical impedance spectroscopy measurements are influenced, as protein deposition modifies charge-transfer resistance and double-layer capacitance.

Despite the scarcity of extensive mechanistic investigations, various mitigating strategies have been documented. These encompass sample dilution, centrifugation, protein precipitation, and the use of size-selective or permselective membranes to reduce interferent access. Surface modification utilizing antifouling polymers, carbon-based nanomaterials, or conductive hydrogels has been extensively implemented to maintain electron-transfer efficiency and diminish surface passivation.

In addition to matrix interference, sensor stability and reusability continue to pose considerable hurdles. Enzyme-based electrodes are susceptible to activity degradation due to repetitive electrochemical cycling, temperature variations, and pH fluctuations. Nanostructured electrodes may experience nanoparticle separation, surface oxidation, or morphological reconstruction during extended operation. From a manufacturing level, attaining consistent electrode patterning, homogeneous nanomaterial deposition, and reproducibility across batches presents substantial obstacles. Although screen-printed and flexible electrodes have significant scalability potential, enhanced uniformity is crucial for commercial and large-scale exploitation in milk quality assessment [104].

While the stability and durability of electrochemical sensors depend on the material characteristics, electrode design, and measurement techniques. Non-enzymatic sensors often provide superior operational stability and reusability compared to enzymatic systems, which are susceptible to environmental variables including pH, temperature, and humidity.

Electrochemical sensors are necessarily sensitive and convenient for analyzing miscellaneous samples as reference methods like colorimetry, fluorimetry, HPLC, GC–MS, and ion chromatography. More importantly, these sensors are cheaper, faster, easier to use, and highly portable, which makes them useful for rapidly monitoring the quality of milk on-site [105].

Addressing major challenges and advantages are essential for transitioning biosensors from the laboratory level to commercial platforms (Table 4). Future investigations must include standardized validation in genuine milk samples, assessments of long-term stability, repeatability analyses across laboratories, and integration with scalable manufacturing techniques.

## 3. Regulatory Thresholds and Analytical Relevance of Biosensor Performance for Milk Adulterant Detection

The effective use of biosensors for monitoring milk adulteration relies on the typical sensitivity parameters such as detection limits and linear ranges with regulatory thresholds or frequently observed adulteration levels. Fresh milk generally has a pH of 6.6–6.8, with variations from this range indicating spoiling, dilution, or chemical adulteration. Both optical and electrochemical sensors consistently detect pH variations with adequate resolution for screening applications [106].

Urea, naturally present in milk at low millimolar quantities, is frequently added illicitly at concentrations exceeding 1–10 mM to artificially augment perceived protein content. Based on the existing literature studies, it is inferred that both enzymatic and non-enzymatic sensors typically attain micromolar or sub-millimolar limits of detection, facilitating identification far below adulteration thresholds [25]. Formaldehyde is banned in milk; when improperly utilized as a preservative, it is generally found at mg per liter (mg L^−1^) quantities. Both optical and electrochemical sensors exhibiting sub-micromolar to micromolar sensitivity are thus analytically recommendable for FA determination [34].

In response to significant food safety occurrences, international authorities limit MEL at minimal levels (mg kg^−1^ range) in dairy products [5]. According to the FSSAI, the permissible amount of MEL in milk for adults is 2.5 ppm, whereas for infants it is 1 ppm. The presence of MEL in milk beyond this limit can cause serious health issues such as renal failure and kidney stones and may also lead to death [107]. The above-discussed colorimetric, fluorometric, and electrochemical sensors comprising detection limits ranging from nanomolar to low micromolar, signifying sufficient sensitivity in relation to regulatory thresholds. NO_3_^−^ and NO_2_^−^ are prohibited as intentional additives in milk inside EU territories and are anticipated to be present only at trace amounts. Electrochemical sensors with LOD ranging from nanomolar to low micromolar are suitable for pollution monitoring needs [108]. Sulfites must be disclosed when present at concentrations of 10 mg kg^−1^ or greater (expressed as SO_2_ equivalents), although the reported sensitivities of the sensor generally remain far below this threshold [109].

Altogether, the above-mentioned biosensors typically demonstrate analytical performance corroborating with regulatory limits for intake of typical milk adulterants regarding precision, portability, and high sensitivity features.

## 4. Long-Term Stability, Reproducibility, and Batch-to-Batch Consistency

Typical parameters such as long-term stability, measurement reproducibility, and batch-to-batch uniformity need to be considered for biosensors against milk adulteration detection, which mainly include disposable paper-based strips and SPEs. For instance, long-term stability is generally assessed by monitoring signal retention, i.e., the percentage of initial response following storage under different conditions, such as temperature, humidity, and light exposure over a period. Previously, numerous investigations evaluated stability using a restricted number of time points; hence, the implementation of more standardized protocols would potentiate the practicality of the biosensors. In this context, enzyme- or aptamer-based sensors may have suffered from instability due to biomolecule degradation, leaching, and loss of activity, while non-enzymatic nanomaterial-based sensors often demonstrate enhanced physicochemical stability, although they may still be susceptible to surface oxidation or aggregation during storage [110].

Reproducibility is often expressed as RSD values among replicate measurements inside a batch (intra-assay) and between independently produced sensors (inter-assay). Regrettably, milk matrices exhibit high variability due to protein and fat fouling and fluctuations in pH, ionic strength, and viscosity, which affect electrode kinetics or optical reading. Since milk is a complex colloidal substance composed of proteins, fat globules, casein micelles, lactose, and inorganic ions, the sensitivity and selectivity of the biosensors may be disrupted. In optical sensors, fat-induced turbidity and protein aggregation can result in light scattering, baseline drift, and inner-filter effects, hence diminishing signal capacity. The adsorption of proteins onto nanomaterials may modify nanoparticle aggregation behavior or diminish fluorescence, impacting analytical performance. Milk proteins and lipids can adhere to electrode surfaces, leading to biofouling, heightened charge transfer resistance, and reduced current response in electrochemical sensors. For example, casein micelles may obstruct electroactive sites, while lipid coatings can hinder electron transfer, especially after prolonged exposures of the sensor. The matrix interference can be mitigated via basic sample dilution using buffer or electrolyte solutions, which is frequently utilized to reduce viscosity and matrix complexity. Furthermore, centrifugation, filtering, or partial defatting might enhance optical visibility and electrochemical stability; however, excessive pretreatment may restrict the analytical performance. Antifouling surface modifications, including polymer coatings, such as Nafion, polyethylene glycol, or zwitterionic layers, have proven effective in reducing nonspecific adsorption in sensors. The microfluidic separation, ratiometric detection, and signal-correction algorithms may further mitigate matrix-induced variability. Therefore, comprehensive assessments across various milk types are still scarce, suggesting that there must be standardized matrix interference studies to facilitate effective and convenient on-site monitoring of milk adulterants [111].

Batch-to-batch consistency continues to be a significant restriction, especially when a sensor is designed through manual drop-casting, uncontrolled drying, or inconsistently dispersed nanomaterials. Conversely, sensors fabricated using screen printing, photolithography, or roll-to-roll processing may provide enhanced design control; nonetheless, they still require validation of electrode uniformity regarding active surface area, resistance, and baseline signal. For better understanding of the feasibility of the optical and electrochemical sensors, subsequent research studies should report (i) *n* ≥ 3–5 batches, (ii) intra-/inter-batch RSD, (iii) storage conditions, and (iv) shelf-life parameters. Moreover, quality-control measures, including internal standards (ratiometric readout), reference channels, antifouling coatings, and standard calibration protocols, may further diminish variability and enhance the consistency of the biosensors [112,113].

## 5. Wearable and IoT-Enabled Biosensors for Continuous Dairy Quality Monitoring

Various adulterants can be detected with increased sensitivity and specificity, which enables the precise detection of fraudulent adulterants to ensure consumer safety. Transforming sensing technologies into wearables and the Internet of Things (IoT) can enhance dairy monitoring. Promising biosensing devices can be developed by employing flexible SPEs, tiny electrochemical transducers, and smartphone-assisted readout systems. The dairy value chain, which includes farms, processing facilities, and distribution systems, could significantly benefit from these measures [114].

Still, some challenges persist, such as sensor miniaturization, fouling resistance, long-term calibration stability, and selective detection within intricate milk matrices abundant in proteins and lipids. To mitigate these constraints, novel platforms such as multi-analyte sensor arrays and hybrid optical-electrochemical sensors are now being investigated. Future biosensors for detecting milk adulteration are anticipated to evolve into wearable devices, smart packaging, and portable sensors. Integrated systems can assess chemical adulterants, protein integrity, microbiological risk indicators, and the overall freshness of milk, thereby improving dairy quality assurance. Altogether, diversified biosensing materials and adaptable wearables progress next-generation dairy monitoring systems [115].

Furthermore, the integration of IoT technology with spectroscopic and various sensor types offers a powerful means for evaluating milk quality and authenticity. Recently, Artificial intelligence (AI) combined with the IoT improves milk quality assurance by analyzing production data, including fat and protein content, and identifying adulteration. This enhances the operational efficiency of dairy operations by delivering real-time milk quality assessments for informed decision-making. For example, if the AI system identifies compositional changes, the process manager can notify standardization procedures for various uses, such as cheese production, quality of protein-fortified products, and economic returns through suitable segregation within the facility. Both IoT and AI technologies can transform milk composition monitoring by using diverse sensors, including spectral, electrochemical, pH, temperature, and humidity sensors, to perpetually assess and gather data on milk quality. IoT devices relay real-time data to a central system, where AI algorithms analyze it to discern patterns, forecast composition, and detect safety concerns [116,117].

## 6. Existing Limitations, Research Needs, and Future Perspectives

Biosensors developed for the detection of adulterants in milk provide significant potential for safeguarding food safety and quality. To achieve widespread market adoption, the commercialization of these biosensors must overcome industrial challenges. The lack of clear regulatory guidelines hinders the biosensors’ effectiveness in assessing food quality. Regulatory bodies mandate comprehensive validation and evidence of reliability prior to allowing the conventional exploitation of these biosensors [118]. Although numerous biosensors demonstrate encouraging outcomes in controlled laboratory environments, obstacles persist in substantiating their efficacy across various laboratories and authentic milk samples. The composition of milk can vary considerably due to factors such as the animal’s diet, breed, and processing methods, which might affect sensor performance. Current research sometimes neglects to sufficiently examine the long-term stability and reproducibility of biosensors, which are essential for guaranteeing consistent outcomes over time. Although biosensors are often described as economical alternatives, the shift to commercial-scale production presents numerous obstacles. Manufacturing processes must ensure uniform quality and performance across batches, a difficulty intensified by the inclusion of delicate nanomaterials or biological elements that may be challenging to produce consistently. Additionally, costs related to sensor calibration, packaging, electrical integration, and quality control augment the overall expenditure, potentially impeding commercial viability [119,120].

Still, there are much improvements that are needed in the development of sensors for the above-mentioned milk adulterants; however, some research gaps still exist in detecting remaining adulterants. For the last decade, food waste (FW) has been transformed into CQDs, yielding favorable economic effects on agriculture and providing environmental advantages. Future research should prioritize overcoming the challenges related to the conversion of FW into eco-friendly CQDs for the detection of multiplex adulterants. Ongoing research into the application of CQDs is anticipated to provide novel CQD-based solutions that enhance food safety and mitigate food loss [16]. Biosensors that can identify analytes in intricate matrices with less cost burden and sufficient portability are in demand. Furthermore, economic factors must be evaluated for the commercialization and scaling of these sensors within the food business and the regulatory bodies tasked with overseeing food quality and safety [121].

Despite notable advancements in biosensor-based detection of milk adulterants, substantial opportunities persist to improve analytical performance and enable practical implementation. Future developments are expected to be profoundly impacted by future digital and data-centric technologies that can address current issues associated with complex food matrices and variable sensing settings.

Machine learning and artificial intelligence are expected to transform next-generation biosensing technologies significantly. Advanced computing methods, such as support vector machines, artificial neural networks, and deep learning algorithms, facilitate efficient signal processing, pattern recognition, and multivariate data analysis. These techniques are especially beneficial for sensor arrays and multichannel systems, where extensive and intricate datasets require precise analysis. Data-driven models can enhance detection reliability and diminish false-positive or false-negative results by adjusting for matrix-induced variability and environmental changes.

A significant focus pertains to the advancement of multiplexed and multi-analyte biosensors. In contrast to traditional single-target assays, these platforms seek to identify various chemical adulterants concurrently, such as urea, MEL, H_2_O_2_, FA, and detergents within a unified analytical process. Sensor arrays and electronic tongue designs provide an effective framework for comprehensive milk quality evaluation, resulting in reduced analysis time, lower operational expenses, and enhanced diagnosis accuracy.

The amalgamation of biosensors with advanced digital technology is anticipated to transform food safety monitoring. Smartphone-assisted readout systems, wireless sensing devices, and cloud-based data processing platforms can expedite decision-making at pivotal junctures within dairy production and distribution chains. In this context, integrating biosensor outputs with blockchain technology provides a robust solution for safe and tamper-proof data management. Blockchain-enabled traceability ensures openness of analytical records, enhances regulatory compliance, and bolsters customer trust through real-time tracking of milk quality information [122,123,124,125,126].

Future research must prioritize long-term sensor stability, reproducibility, standardized validation techniques, and scalable manufacturing strategies to fully capitalize on these technical advancements. Addressing these practical obstacles is crucial for closing the divide between laboratory-scale demonstrations and commercial implementation. Ongoing multidisciplinary collaboration among materials scientists, analytical chemists, data scientists, and food safety stakeholders will be essential for transforming biosensors into reliable instruments that can enhance next-generation milk safety surveillance systems.

Future research should rely on progressing globally standardized validation procedures implemented by regulatory bodies. Simple, robust, and scalable sensors will enhance economic feasibility, and regulatory authorities may establish approval standards that mitigate costs and reduce the time required for industrial implementation. More importantly, achieving an ideal balance between cost and performance is essential in advancing biosensors that are both affordable and reliable for evaluating dairy quality. Biosensors for detecting milk adulterants show promising results. However, it is important to address regulatory, validation, and economic issues so that these technologies can shift from the lab to the market.

## 7. Conclusions

Rapid, reliable monitoring of milk quality is becoming increasingly essential as milk adulteration practices evolve and dairy supply chains expand. Optical and electrochemical biosensing platforms have emerged as powerful alternatives to conventional laboratory assays due to their sensitivity, portability, and capability for real-time, on-site analysis. Recent advances in nanofabrication, microfluidics, quantum cascade spectroscopy, and hybrid biointerfaces have enabled the detection of key milk adulterants, such as NO_3_^−^, NO_2_^−^, and SO_3_^2−^ ions, FA, MEL, pH, and urea, with unprecedented precision. These developments are not only improving quantification of nutritional markers but also enabling early identification of fraudulent dilution, protein substitution, and adulterant detection. The translation of these technologies into wearable and IoT-enabled devices marks a pivotal shift in dairy safety monitoring. Integration of flexible electrodes, smartphone interfaces, cloud-based analytics, and AI-driven pattern recognition is transforming sensors into decentralized quality control tools suitable for farmers, processing industries, and consumers [127]. While challenges remain in sensor miniaturization, fouling resistance, long-term calibration stability, and selective detection within complex milk matrices, multi-sensor fusion and smart algorithmic compensation are promising solutions.

The increasing need for rapid and dispersed food safety evaluations has accelerated the development of portable electrochemical biosensors for detecting milk adulteration. An et al. developed a compact and integrated sensing device for the detection of MEL in milk by integrating an SPCE with a low-power electrochemical workstation. Ferrocenylglutathione (Fc-ECG) serves as an electron transfer mediator, enhancing the signal amplification of the intrinsically weak electrochemical response of MEL. Microcontroller-based detection devices enhance portability for performing DPV analysis. The analytical performance of the portable sensors is highly correlated with that of conventional electrochemical sensors in terms of reliable sensitivity, selectivity, repeatability, and stability in real milk samples. These portable electrochemical biosensors establish a scientific basis for prospective integration with wearable devices, smart packaging, and IoT-enabled monitoring systems [128].

In another study, authors developed a smartphone-assisted, paper-based colorimetric biosensor for the detection of urea adulteration in milk [25]. To facilitate portability, the sensing material is applied to paper-based substrates, and the analytical signal is quantified by smartphone-based RGB picture analysis. Performance assessment of authentic milk samples exhibited dependable detection capabilities, with enhanced analytical response after minimal sample pretreatment. Several similar studies have emphasized the recent progress in the development of miscellaneous portable sensors [129,130,131,132,133,134,135].

Future research on milk adulteration detection could benefit from increased emphasis on multiplexed biosensing approaches that enable the concurrent determination of multiple adulterants using a single biosensor. Hence, the continuous development of cost-effective and portable biosensing devices is essential for efficient on-site testing. Future initiatives must prioritize enhancing operational stability, resistance to matrix interference, and repeatability.

In the coming years, next-generation sensors for the determination of milk adulterants are likely to evolve into self-powered, real-time, adaptable wearable platforms integrated with packaging, handheld scanners, or livestock-attached tags. Such systems will not only authenticate protein composition but also monitor cold-chain integrity, microbial contamination risk, and overall milk freshness. Ultimately, the convergence of optical and electrochemical sensing innovations with wearable technology represents a transformative step toward democratized food safety, traceable dairy supply chains, and global consumer protection in an era of increasing nutritional needs.

## Figures and Tables

**Table 1:** Summary of colorimetry-based sensors for milk adulterants detection.

Materials	Adulterants	Sample Type	Analytical Method	Sensor Format	Linear Range	Detection Limit	Reference
HNT/Urs	Urea	Water and Milk	Colorimetric	Optical sensing	0.5–100 mM	0.2 mM and 2.54 mM	[25]
MnO_2_ NSs	H_2_O_2_ and Lactic acid	Milk	Colorimetric	Optical sensing	0–0.1 mM	0.72 mM and 0.75 mM	[29]
TiOSO_4_/Guar gum	H_2_O_2_	Milk	Colorimetric	Optical sensing	0–1.47 mol L^−1^	5.88 mM	[30]
AuNPs and AgNPs	MEL	Milk	Colorimetric	Optical sensing	0.016–0.16 mM and 0.016–0.14 mM	13.33 μM and 5.08 μM	[31]
Para-dimethylaminobenzaldehyd (p-DMAB) solution	Urea, Starch, Salt, Detergent, H_2_O_2_, Soap, NaHCO_3_	Milk	Colorimetric	Optical sensing	0.05–1.0% (*v*/*v*)	0.05–0.2% (*v*/*v*)	[32]
Citrate- AgNPs	Urea	Milk	Colorimetric	Optical sensing	1–15 mM	5.56 μM	[33]
PVC/NPOE	FA	Milk	Colorimetric	Optical sensing	0–203 μM	6.7 μM	[34]
AgNPs	MEL	Milk	Colorimetric	Optical sensing	0.00079–7.93 mM	0.00079 mM	[35]
Suc-Ag NPs	MEL	Water	Colorimetric	Optical sensing	0.1–1.2 μM	0.01 μM	[36]
Metal Nanoclusters	FA, H_2_O_2,_ and Sodium hypochlorite (NaClO)	Milk	Colorimetric	Fluorescence	0–1440 mM, 0–1180 mM, and 0–590 mM	18.0 mM, 1.48 mM, and 2.94 mM	[37]

**Table 2:** Summary of fluorescence-based sensors for the detection of milk adulterants.

Materials	Adulterants	Sample Type	Analytical Method	Sensor Format	Linear Range	Detection Limit	Reference
Nanoclusters	FA, H_2_O_2,_ Sodium hypochlorite (NaClO)	Milk	Fluorescence	Optical sensing	0–1440, 0–1180, and 0–590 mM	18.0, 1.48, and 2.94 mM	[37]
CQDs	pH and Urea	Milk	Fluorescence	Optical sensing	1–14 (pH) and 0–30 mM (Urea)	1.6 mM and 1.9 mM	[46]
R-CDs/MR and NIR-CDs/Cu^2+^	Urea	Milk	Fluorescence IFE	Optical sensing	0.001–8.0 mM	0.28 μM	[47]
NFD	FA	PBS (pH 7.4)	Fluorescence PET	Optical sensing	30–100 μM	0.95 μM	[49]
InP/ZnS QDs/NAHN probe	FA	Water	Turn-on Fluorescence (PET)	Optical sensing	0–200 μM	0.623 μM	[50]
RBNA	FA	Ethanol/tris-HCl solution (*v*/*v* = 3/7, pH 7.4)	Turn-on Fluorescence (PET)	Optical sensing	0–120 μM	0.21 μM	[51]
MoSe_2_/SH QDs	MEL	HEPES buffer (pH 7.4)	Turn-on Fluorescence [Fluorescence resonance energy transfer (FRET)]	Optical sensing	0–1000 nM	27.7 nM	[52]
Acrylated citric acid (ACA)-based polymeric membrane sensor	MEL	Britton Robinson (BR) buffer (pH 6.0)	Turn-off Fluorescence	Optical sensing	3.96–79.3 nM	0.232 nM	[53]
Tb-GQDs	MEL	Water	Turn-off Fluorescence (Static quenching)	Optical sensing	0–5.0 μM	0.31 μM	[54]
PEI-AgNPs	MEL	pH 6.0 (0.1 M Tris-HCl)	Turn-off Fluorescence (Hydrogen bonding)	Optical sensing	0.16–56 μM	0.132 μM	[55]
N-CQDs-Fe^3+^	MEL	Water	Turn-on Fluorescence (Coordination interaction)	Optical sensing	2.0–290 μM	0.66 μM	[56]
DNA/AgNCs + Hg^2+^	MEL	PBS (pH 7.0)	Turn-on Fluorescence (Coordination interaction)	Optical sensing	0.2–4.0 μM	0.1 μM	[57]
Polyelectrolyte (CPE)-(P1-AgNPs)	MEL	Na_2_CO_3_–NaHCO_3_ buffer (pH 9.0)	Ratiometric fluorescence	Optical sensing	0.1–1.0 nM	0.1 nM	[58]

**Table 3:** Summary of electrochemical sensors for the detection of milk adulterants.

Materials	Adulterants	Sample Type	Analytical Method	Sensor Format	Linear Range	Detection Limit	Reference
Fe_3_O_4_/MWCNT/PANI-Nafion/Urs/GCE	Urea	PBS (pH 7.4)	CV, DPV, and CA	GCE	1.0–25.0 mM	67 μM	[69]
Functionalized-GNPlts/GNDs/Urs/SPE	Urea	Water	Direct current voltage (IV)	SPE	1.67–15.0 mM	83.3 μM	[70]
Nf/PANI/CuF/Urs/GCE	Urea	PBS (pH 7.0)	DPV	GCE	0.5–45.0 μM	0.17 μM	[71]
AuNPs/GO/Urs/GCE	Urea	PBS (pH 7.4) and Milk	DPV	GCE	0.83–15.0 mM	6.2 ± 2.0 μM and 8.2 ± 1.8 μM	[72]
La-doped CoFe LDH@rGO	Urea	KOH solution (0.1 M)	CV and CA	CPE	0.001–23.5 mM	0.33 ± 0.11 μM	[75]
CuO/Co_3_O_4_@MWCNTs/SPE	Urea	KOH solution (0.1 M)	CV and EIS	SPE	10 mM–1.0 pM	0.223 pM	[76]
NiS/GO/MGCE	Urea	KOH solution (1.0 M)	CV	GCE	0.1–1.0 mM	3.79 μM	[77]
Fe_3_O_4_@SiO_2_ NPs	NO_2_^−^ and SO_3_^2−^	PBS (pH 7.0)	DPV	GCE	0.01–1.0 mM and 0.1–8.0 mM	3.33 μM and 31.57 μM	[83]
Au NDs@FSPCEs	NO_2_^−^	PBS (pH 4.5)	CA	FSPCE	0.02–5.8 μM	1.0 nM	[84]
Ni-Cu(PDA)MOF/CNF	BPA	PBS (pH 7.0)	DPV	GCE	1–150 μM	75 nM	[93]
CeO_2_@C-QAs	MEL	PBS (pH 3.6)	DPV	GCE	20 nM–5.0 μM	2.19 nM	[97]
CC/CeO_2_/CNPs	MEL	PBS (pH 3.6)	DPV	GCE	0–30 nM	0.274 nM	[98]
CuO-CNT NCs/Nafion/GCE	MEL	PBS (pH 8.0)	Direct current voltage (IV)	GCE	0.05–0.5 nM	0.27 nM	[99]
rGO-PPYPPA	MEL	PBS (pH 7.4)	CV and EIS	GCE	10 pM–10 mM	12 pM	[100]

CV: Cyclic voltammetry, DPV: Differential pulse voltammetry, CA: Chronoamperometry, GCE: Glassy carbon electrode, SPE: Screen-printed electrode, CPE: Carbon paste electrode, EIS: Electrochemical impedance spectroscopy, FSPCE: Flexible screen-printed carbon electrode.

**Table 4:** Comparative overview of different biosensor platforms for milk adulterant detection.

Sensor Type	Sensitivity	Selectivity	Cost	Instrumentation	Portability	Major Advantages	Major Limitations
Colorimetric	Moderate	Moderate	Low	Minimal (visual or smartphone)	Excellent	Simple operation; Rapid response; Low cost; Suitable for on-site screening.	Limited sensitivity; Matrix turbidity interference; Subjective color interpretation.
Fluorimetric	High to very high	High	Moderate	UV/optical source	Good	Low detection limits; High signal amplification; Compatibility with imaging systems.	Autofluorescence interference; Photobleaching; Optical instability in milk.
Electrochemical	High	High	Moderate	Portable potentiostat	Good/excellent	Quantitative accuracy; Fast response; Miniaturization capability.	Electrode fouling; Reproducibility issues; Surface instability.

## Data Availability

No new data were created or analyzed in this study. Data sharing is not applicable to this article.
